# Supplementary material for: Limited overlap between genetic effects on disease susceptibility and disease survival
Source: Nat Genet. 2025 Sep 30;57(10):2418–26. doi: 10.1038/s41588-025-02342-8 (PMC12513829; doi:10.1038/s41588-025-02342-8)
Supplement: Supplementary file 1 — Supplementary Note and Figs. 1–20. [file 41588_2025_2342_MOESM1_ESM.pdf]

nature genetics

Supplementary information https://doi.org/10.1038/s41588-025-02342-8

Limited overlap between genetic effects on disease susceptibility and disease survival

In the format provided by the authors and unedited

Supplementary Note

Simulation to explore the impact of index event bias

To investigate the impact of index event bias, we carried out simulations under a simple liability threshold model where we defined the liability to disease susceptibility under a polygenic risk model as random variable S

Total genetic effect on

Vector of individual genotype

$$S = \beta_S^T g + \epsilon_S$$

Environmental noise on

Variant effect on susceptibility

where g is the random vector for standardised genotype and β_{gi} is the random vector of their effect sizes on the diagnosis liability, ϵ_S is the zero mean residual independent to $\beta_S^T g$.
Next, we defined liability to disease progression as a random variable P which depends both on the causal effect of diseases susceptibility (c) and some unique genetic effect on disease progression ($\beta_P^T g$)

Effect of susceptibility liability on progression

$$P = \beta_P^T g + cS + \epsilon_P$$

Environmental noise on

Unique genetic effect on

We define the heritability of disease susceptibility (h_{sus}) and *unique genetic components* of disease progression (h_{prog}) as:

$$h_{sus} = \frac{Var(\beta_S^T g)}{Var(S)}$$

$$h_{prog} = \frac{Var(\beta_P^T g)}{Var(P)}$$

Last, we define ρ as the correlation of the polygenic effect between disease susceptibility and disease progression:

$$\rho = \frac{Cov(\beta_P^T g, \beta_S^T g)}{\sqrt{Var(\beta_P^T g) Var(\beta_S^T g)}}$$

We carried out a simulation based on the genome of chromosome 21 (containing 111,212 HM3 SNPs) for 10,000 synthetic European individuals created using Hapgen2 (Su et al., 2011). In the simulation, we fixed heritability of disease susceptibility at h_{sus} = 0.2, and impact of susceptibility liability on disease progression liability at *c* = 0.3. We further fixed the compositions of causal SNPs for each of these two endpoints so that 0.001 of total SNPs (ie. around 110 in this case) have direct effect on disease susceptibility and 0.001 have direct effect on progression. We changed the proportion of overlap between these two genetic components so that 25%, 50%, 75% of the causal SNPs were shared between susceptibility and progression. To decide effect size for each causal SNP *i*, we first drew a base effect independently from a standard univariate normal distribution

$$\beta_{i,base} \sim N(0, 1)$$

and multiply it to the square root of heritability this SNP accounts for to get its final effect size. Causal SNPs shared by susceptibility and progression were simulated to have same base effect on susceptibility and progression so that expected correlation of overall polygenic effects between two endpoints ρ will approximately correspond to the proportion of shared causal SNPs, in this case ρ = 0.25, 0.5 and 0.75. We further vary heritability of disease progression h_{prog} = 0.005, 0.1, and 0.2.

Under each simulation setup, we run standard GWAS correcting for top 10 PCs for both susceptibility and progression liability. Note that just like we added age of diagnosis as a covariate in our empirical mortality GWAS, in the progression GWAS, we also correct for susceptibility liability. Subsequently, we clump the GWAS results using plink (Purcell et al., 2007) under parameters --clump-p1 5e-8 --clump-r2 0.5 --clump-kb 250 to extract independent genome-wide significant loci from each GWAS.

Impact of index event bias and Slope-Hunter-like adjustment

To investigate the impact of index event bias, we think the most direct way would be to compare the underlying simulated SNP effects to observed effects from GWAS for disease progression. Recall that effect size for each causal SNP *i* is a standard normal variable multiplied by square root of its heritability, which can be expressed as

$$\beta_i = \sqrt{\frac{h}{\mathrm{Var}(\sum_{i=1}^{n} \beta_{i,base}\, g_i)}}$$

, where β_i is the underlying causal effect simulated, h is the endpoint heritability, g_i is the genotype of causal SNP *i,* and *n* is the total number of causal SNPs for the endpoint. The same equation applies to both susceptibility and progression causal genetic effects.

In this experiment as we are investigating the impact of index event bias, on top of shared polygenic effects, we introduced another component *u* to account for any other shared non-genetic risk factor between the two. Same as previous experiment, heritability of disease susceptibility and impact of susceptibility liability on disease progression liability were still fixed as h_{sus} = 0.2 and c = 0.3. For this experiment, we further fixed the heritability of disease progression at h_{prog} = 0.005. We vary ρ = 0.25, 0.5 and 0.75, and contribution of the non-genetic component on variance of susceptibility and progression liability among Var_u = 0, 10%, or 20%.

Under each setup, we ran GWAS on disease susceptibility and progression as described before, and for all progression causal variants, we plotted simulated SNP effects against SNP effects observed from the progression GWAS. We examined the residual sum of squares (rss) for the points around function y = x.

Furthermore, based on the theory behind of Slope-Hunter, we applied adjustment on SNPs that suffer from index event bias through a procedure described as below:

1. Extract all susceptibility specific causal SNPs and regress their observed effect sizes from susceptibility GWAS against progression GWAS to obtain the correction factor *b*.
2. For each causal SNP *i* shared between susceptibility and progression, compute the corrected progression genetic effect $\widehat{\beta_{i,prog}}$ as below

$$\widehat{\beta_{i,prog}} = \widehat{\beta_{i,prog}}^{*} - b\widehat{\beta_{i,prog}}$$

, where $\widehat{\beta_{i,prog}}^{*}$ is the observed effect for SNP *i* from the conditioned progression GWAS, and $\widehat{\beta_{i,sus}}$ is the observed effect for SNP *i* from the susceptibility GWAS. Note that this experiment

may demonstrate the utility of Slope-Hunter-like correction in a nearly "perfect" scenario, where the classification of SNPs (susceptibility specific, progression specific, shared or no effect in either) is given. In practice, a Bayesian or comparable approach needs to be applied for posterior variant group assignment, which can result in worse performance than shown in this manuscript. As a comparison, we show in the same plot the corrected variants effect sizes against the simulated underlying effects and examined rss. See **Supplementary Figure** 20 for results.

Note the previous experiment shows impact of index event bias and correction on all underlying causal variants for the progression, whereas in practice, such information is not a given. Therefore, subsequently we tried to examine the real impact of Slope-Hunter-like correction on observed results from GWAS under one of the conditions where the most severe index event bias could be observed (h_{prog} = 0.005, Var_u = 20%, ρ = 0.5). We chose ρ = 0.5 rather than ρ = 0.75, where more causal variants are shared, so that more susceptibility specific SNPs were available for correction factor (b) estimation and a more accurate estimate could be achieved. For this experiment, we first ran progression and susceptibility GWAS respectively as mentioned before and clumped their results to identify independent signals for each. Then using susceptibility specific SNPs, we fitted the correction factor and applied correction on all SNP effects in the progression GWAS. We also corrected for the standard errors as mentioned in (Mahmoud et al., 2022) and recomputed the p-values. Mindful of the difference from the previous experiment, that correction was not only applied on shared causal SNPs but all variants, as that would be what is done empirically. Note that here we still provided causal information for susceptibility specific variants to fit the correction factor, which was again a rather ideal use case for the method. None of the SNPs became genome-wide significant in progression GWAS after correction (**Supplementary Table** 11).

Data and resources

FinnGen

Genotyping and quality control

FinnGen consists of prospectively recruited samples and a series of legacy cohorts with genotypes already available (*FinnGen Project | FinnGen*, n.d.). Prospective samples were genotyped using the ThermoFisher Axiom custom array which tags a total of 655,973 variants. Genotype calling was performed using the Array Power Tools software. Legacy cohorts were genotyped using various Illumina arrays and genotype calling was performed using either GenCall or zCall algorithms.

For both prospective and legacy cohorts the following quality control metrics were used.

Samples were removed if:

- Pihat was > 0.9 and the samples were not monozygotic or replicates
- There was a discrepancy between reported sex and genetically determined sex (F-value ≤ 0.3 for females and ≥ 0.8 for males)
- Missingness was ≥ 5%
- Heterozygosity was ±4 standard deviations from the population average
- Pihat was > 0.1 with 14 or more samples
- Samples were ±4 standard deviations away from the population average according to the first two genetic principal components.

Samples were tagged should there be evidence of a mendelian error or contain replicate samples with over 50,000 discrepancies.

Variants were removed if:

- The variant failed the Hardy-Weinberg Equilibrium test (p-value < 10-6)
- The variant had a call rate < 98%

Imputation

Pre-phasing was performed using Eagle 2.3.5 (Loh et al., 2016) and samples were imputed using the SiSu v3 imputation reference panel. This reference panel is specific to the Finnish population, containing high-coverage (25-30x) whole-genome sequencing data from 3,775 Finns and 16,962,023 variants with minor allele count ≥ 3. After imputation, 16,387,711 variants were imputed with high quality (INFO > 0.6).

Ancestry assignment

Firstly, the FinnGen samples were combined with the 1000 genomes phase 3 dataset. Genetic principal components were calculated using a subset of 49,451 pruned SNPs. Aberrant (Bellenguez et al., 2012) was used to identify and remove samples that deviated from the main cluster. A probability of belonging to either a North-Western European or Finnish population was calculated by firstly performing PCA with individuals belonging to these ancestries from 1000 genomes data. FinnGen samples were then projected onto this PCA space and Mahalanobis distances calculated for each sample against each of the two ancestries. Samples were retained if there was ≥ 95% probability of belonging to the Finnish ancestry cluster.

Ethics statement

Patients and control subjects in FinnGen provided informed consent for biobank research, based on the Finnish Biobank Act. Alternatively, separate research cohorts, collected prior the Finnish Biobank Act came into effect (in September 2013) and start of FinnGen (August 2017), were collected based on study-specific consents and later transferred to the Finnish biobanks after approval by Fimea (Finnish Medicines Agency), the National Supervisory Authority for Welfare and Health. Recruitment protocols followed the biobank protocols approved by Fimea. The Coordinating Ethics Committee of the Hospital District of Helsinki and Uusimaa (HUS) statement number for the FinnGen study is Nr HUS/990/2017.

The FinnGen study is approved by Finnish Institute for Health and Welfare (permit numbers: THL/2031/6.02.00/2017, THL/1101/5.05.00/2017, THL/341/6.02.00/2018, THL/2222/6.02.00/2018, THL/283/6.02.00/2019, THL/1721/5.05.00/2019 and THL/1524/5.05.00/2020), Digital and population data service agency (permit numbers: VRK43431/2017-3, VRK/6909/2018-3, VRK/4415/2019-3), the Social Insurance Institution (permit numbers: KELA 58/522/2017, KELA 131/522/2018, KELA 70/522/2019, KELA 98/522/2019, KELA 134/522/2019, KELA 138/522/2019, KELA 2/522/2020, KELA 16/522/2020), Findata permit numbers THL/2364/14.02/2020, THL/4055/14.06.00/2020, THL/3433/14.06.00/2020, THL/4432/14.06/2020, THL/5189/14.06/2020, THL/5894/14.06.00/2020, THL/6619/14.06.00/2020, THL/209/14.06.00/2021, THL/688/14.06.00/2021, THL/1284/14.06.00/2021, THL/1965/14.06.00/2021,THL/5546/14.02.00/2020, THL/2658/14.06.00/2021, THL/4235/14.06.00/2021, Statistics Finland (permit numbers: TK-53-1041-17 and TK/143/07.03.00/2020 (earlier TK-53-90-20) TK/1735/07.03.00/2021, TK/3112/07.03.00/2021) and Finnish Registry for Kidney Diseases permission/extract from the meeting minutes on 4th July 2019.

The Biobank Access Decisions for FinnGen samples and data utilized in FinnGen Data Freeze 10 include: THL Biobank BB2017_55, BB2017_111, BB2018_19, BB_2018_34, BB_2018_67,

BB2018_71, BB2019_7, BB2019_8, BB2019_26, BB2020_1, BB2021_65, Finnish Red Cross Blood Service Biobank 7.12.2017, Helsinki Biobank HUS/359/2017, HUS/248/2020, HUS/150/2022 § 12, §13, §14, §15, §16, §17, §18, and §23, Auria Biobank AB17-5154 and amendment #1 (August 17 2020) and amendments BB_2021-0140, BB_2021-0156 (August 26 2021, Feb 2 2022), BB_2021-0169, BB_2021-0179, BB_2021-0161, AB20-5926 and amendment #1 (April 23 2020)and it´s modification (Sep 22 2021), Biobank Borealis of Northern Finland_2017_1013, 2021_5010, 2021_5018, 2021_5015, 2021_5023, 2021_5017, 2022_6001, Biobank of Eastern Finland 1186/2018 and amendment 22 § /2020, 53§/2021, 13§/2022, 14§/2022, 15§/2022, Finnish Clinical Biobank Tampere MH0004 and amendments (21.02.2020 & 06.10.2020), §8/2021, §9/2022, §10/2022, §12/2022, §20/2022, §21/2022, §22/2022, §23/2022, Central Finland Biobank 1-2017, and Terveystalo Biobank STB 2018001 and amendment 25th Aug 2020, Finnish Hematological Registry and Clinical Biobank decision 18th June 2021, Arctic biobank P0844: ARC_2021_1001.

UK Biobank

Genotyping and quality control

UK Biobank participants were genotyped by two genotyping arrays: The UK Biobank Lung Exome Variant Evaluation (UKBiLEVE) Axiom array was used to genotype 49,950 participants. The remaining 438,427 participants were genotypes using the Applied Biosystems UK Biobank Axiom Array. Principal Component Analysis (PCA) was performed on the genetic data and centralised quality control (QC) on variants was performed on individuals identified to belong to the largest cluster (N=463,844) according to Aberrant - an unsupervised clustering algorithm (Bellenguez et al., 2012). Variants were assessed for evidence of allele frequency variation across batch, plate, sex or array and that genotypes were largely consistent with Hardy-Weinberg Equilibrium expectations (all p-value thresholds < 10^{-12}). If a variant failed one or more tests within a given batch it was set to missing. See (UK Biobank, 2015) for more detailed information on testing.

Imputation

For 487,442 individuals, imputation was performed using the IMPUTE4 (Howie et al., 2009) software. Genetic variation from the Haplotype Reference Consortium (HRC) (McCarthy et al., 2016) and merged UK10K+1000 Genomes (1000 Genomes Project Consortium, 2015) were used as a reference panel. Single Nucleotide Polymorphisms (SNPs) were only included in the final imputation if they were present in both reference panels, giving a total of 96,959,328 SNPs.

Ancestry assignment

Ancestry assignment uses methodology and scripts from GenoPred (*Prediction within Ancestral Diversity*, n.d.). Individuals were stratified into one of five super populations African (AFR), American (AMR), South Asian (SAS), East Asian (EAS) and European (EUR). The 1000 Genomes data (1000 Genomes Project Consortium, 2015) acted as a reference given the individuals are known to belong to one of the 5 super populations. Only unambiguous SNPs also present in both the HapMap3 consortium (Gibbs et al., 2003) and the imputed UK Biobank data were retained for PCA. SNPs within both the reference (1000 Genomes) and target (UK Biobank) samples underwent quality control such that the minor allele frequency (MAF) > 5%, variant missingness > 2% and Hardy-Weinberg Equilibrium p-value > $1e^{-6}$. 467,970 autosomal SNPs remained following QC and were in the intersection of the reference and target samples. Regions with long range linkage disequilibrium were excluded and independent SNPs (SNPs greater than 1000kb apart and $r^2 < 0.2$) retained. PCA was then

performed in the reference sample using PLINK v2 (Purcell et al., 2007) and a multinomial elastic-net regression was trained using 5-fold cross validation, super population as the outcome and the first 10 PCs as covariates. PCs from the target sample were then projected into the reference space and prediction on super population made. Classifications were made according to the super population with the greatest probability. To be classified the max probability must be over 0.5, otherwise it was set to missing.

PCA was performed using a random subset of 1000 individuals per super population and PC's from the rest of the super population sample projected onto this space. Distances from the centroid were calculated and outliers removed. Outliers were classified as having a distance > 75 percentile + 30*Interquartile Range. Following within-ancestry QC, 8,381, 1,063, 2,393, 447,332 and 9,435 individuals were allocated to AFR, AMR, EAS, EUR and SAS super populations respectively.

<u>Estonian Biobank</u>

Genotyping and quality control

Estonian BioBank (EstBB) samples were genotyped with 4 sub-versions of Infinium Global Screening Array-24. Samples with less than 95% call-rate were excluded. Sample sex recorded in the EstBB database was compared with genetic sex. Samples with sex mismatch were further inspected for sex chromosome abnormalities (X0, XXY, etc.), and samples with confirmed database vs genetic sex mismatch were excluded. In total, 202 910 individuals passed sample quality control. SNP quality control was performed by excluding: (a) all SNPs with less than 95% call-rate, (b) SNPs showing more than 5% AF difference from the AF mean estimated using all genotyping batches with more than 10 000 samples per batch, (c) SNPs with Illumina GenTrain score < 0.6 or cluster separation score < 0.4 in any genotyping batch, (d) autosomal SNPs with HWE exact test p-value < 1e-4. In total, approximately 328K autosomal and X-chromosome SNPs with MAF > 1% passed quality control and were used in the imputation. All the variants were processed on the human genome assembly GRCh37.

Imputation

Imputation was performed using a local Estonian imputation reference panel made of 2056 WGS samples. Genotypes were pre-phased with Eagle v2.4.1 and imputed with Beagle 5.1 using default parameters. Multiallelic positions were excluded from imputation output. In total, 39 546 641 variants were used in the study.

Ancestry assignment

EstBB samples were combined with the 1000 genomes phase 3 dataset for ancestry analysis. Genetic principal components were calculated using a subset of quality controlled and pruned genotyped SNPs. This was further used to identify and remove samples that deviated from the main cluster via visual inspection. In total, 481 non-european ancestry individuals based on principal components were excluded from the analysis.

<u>Genomics England</u>

Genotyping and quality control

Genome sequencing was performed in DNA samples from 78,195 individuals using Illumina HiSeq X systems (150bp paired-end format). Reads were aligned using the iSAAC Aligner (version 03.16.02.19) and small variants were called using Starling Small Variant Caller (version 2.4.7). Samples were aligned to the Homo Sapiens NCBI GRCh38 assembly with decoys.

Aggregation of single-sample gVCFs was performed using the Illumina software gVCF genotyper (version 2019). Variant normalisation and decomposition were implemented by vt

(version 0.57721). Genomic annotation and calculation of allele statistics were performed using Ensembl VEP and bcftools respectively. The multi-sample VCF dataset (aggV2) was then split into 1,371 roughly equal chunks to allow faster processing. Only variants that passed all provided site quality control criteria were processed.

Imputation

The WGS genotypes (~722M variants) were filtered to a variant base list used for PGS model generation, which includes 18,421,839 variants. (For further information on how the variant list was derived see: https://research-help.genomicsengland.co.uk/pages/viewpage.action?pageId=72351761)

Genotypes were phased and imputed using the 1000G reference panel (v5a) which was lifted-over from GRCh37 to GRCh38 using cross-map.

Ancestry assignment

The genetic ancestry of the patients was estimated using a random forest classifier and data from 1000 genomes project phase 3 (1KGP3) dataset. Firstly, all unrelated samples from the 1KGP3 were selected and 188,382 HQ SNPs were subsetted. After filtering for MAF > 0.05 in 1KGP3 (and GE data), the first 20 PCs were calculated using GCTA and the aggV2 data were projected onto the 1KGP3 PC loadings. The random forest model to predict ancestries was trained based on:

A. First 8 1KGP3 PCs
B. set Ntrees = 400
C. Train and predict on 1KGP3 Admixed American, African, East Asian, European, and South Asian super-populations.

Individuals were assigned for any one ancestry with a probability of > 0.8.

<u>Genes and Health</u>

Genotyping and quality control

We used the latest 2021 July GNH data release including 44,190 individuals (26,537 British-Bangladeshi, 17,653 British-Pakistani). Genotyping was performed on DNA samples from saliva, using the Illumina Infinium Global Screening Array v3, which contained 730,059 variants. GenomeStudio from Illumina was used to perform clustering and initial quality control on the genotype data. Variants were removed if they had low call rate, or were tagging structural variants, a positive HetExcess > 0.03, Hardy-Weinberg equilibrium P-value < 1.0×10^{-6}, cluster sep <0.57, or automated clustering (GenTrain) score <= 0.7. A total of 637,829 variants remained with call rates of > 0.992 for female samples and > 0.995 for male samples (including X and Y chromosomes). Sample exclusion criteria included duplicate GSA genotypes that should not be sample duplicates, samples that should be duplicated but have not matching GSA genotypes, and a few late withdrawals of consent. Only chip genotyped samples with valid NHS numbers were preserved. When two chip genotype samples with the same NHS number were found, the samples with the highest call rate were retained.

Imputation

Monomorphic SNPs, non-ACGT, palindromic (A/T, T/A, C/G, G/C), and chr Y variants were excluded. Variants were evaluated by TOPMed QC to obtain SNPs that required strand flipping (performed in plink). Furthermore, variants with MAF<0.0001 were excluded. The TOPMed-r2 Minimac4 Imputation Server (version 1.5.7, <u>https://imputation.biodatacatalyst.nhlbi.nih.gov/#!pages/home</u>), created by the University of Michigan, was subsequently used to impute the genotypes. Rsq filter (imputation quality) of 0.3 was applied within the Imputation Server.

Ancestry assignment

A total of 44,396 individuals and 355,862 directly genotyped variants (retaining only autosomal variants, MAF>0.01, call rate >99% and those passing HWE in declared Bangladeshi individuals) were used with the KING software to estimate pairwise relationship up to 4 degrees. PCA was performed on GNH unrelated individuals, projecting related individuals into the PC, to obtain 50 PCs for all GNH samples. For the ancestry assignment, we used a reference cohort consisting of 3,433 individuals from 1000G and HGDP. A PCA up to 50 PCs was performed on the reference set (3,433 individuals and 104,552 variants) and subsequently the GNH samples were projected into the reference PCA. Using UMAP with 7 PCs, we genetically inferred Bangladeshi and Pakistani individuals and excluded 76 non South Asian outliers and 130 South Asian outliers (not falling into the main clusters).

Generation Scotland

Genotyping and quality control

Generation Scotland (GS) consists of ~24,000 individuals from across Scotland aged between 18-99 years. Phenotypic data were obtained at baseline along with whole blood samples for DNA quantification. Disease outcomes were ascertained through linkage to primary (GP) and secondary (hospital) healthcare records.

Genotype data was assayed for 20,195 participants in two batches with 9,863 participants in the first batch and the remainder in the second. The genotyping was performed using the Illumina HumanOmniExpressExome-8 v1.0 BeadChip and the Illumina HumanOmniExpressExome-8 v1.2 BeadChip, respectively. Individuals or SNPs with a low call rate (<98%) and SNPs with Hardy-Weinberg p-value<$1x10^{-6}$ were removed. Mendelian errors were removed by setting the individual-level genotypes at erroneous SNPs to missing.

Imputation

Genotyped data were imputed using the HRC panel v1.1 (McCarthy et al., 2016). Autosomal haplotypes were checked to ensure consistency with the reference panel (strand orientation, reference allele, position. Pre-phasing was performed using Shapeit2 v2r837 (O'Connell et al., 2014) using the Shapeit2 duohmm option11 (O'Connell et al., 2014) and cohort family structure in order to improve imputation quality (O'Connell et al., 2014). Variants with low imputation quality (INFO<0.4) as well as monogenic variants were removed from the imputed set resulting in 24,111,857 variants for downstream analysis.

Ancestry assignment

Ancestry outliers were removed from the dataset. These were defined as individuals who were more than six standard deviations away from the mean in a principal component analysis of GS merged with 1092 participants from the 1000 Genomes Project (1000 Genomes Project Consortium, 2015).

Dana Farber

Genotyping and quality control

DNA samples were processed from the whole blood and genotyped on either the Illumina Multi-Ethnic Genotyping Array (MEGA), the Expanded Multi-Ethnic Genotyping Array (MEGA Ex) array, or the Multi-Ethnic Global (MEG) BeadChip (Bien et al., 2016). All germline samples were imputed to the Haplotype Reference Consortium (HRC) reference panel (McCarthy et al., 2016) and then restricted to ~1.1 million HapMap3 variants that typically exhibit high imputation accuracy across genotyping platforms and uniformly tag common SNP variation (Finucane et al., 2015). Small indels were not available in the HRC reference panel due to sequencing ambiguity, and we additionally imputed small indels into the germline genotyped

data using the 1000 Genomes Phase 3 reference panel (1000 Genomes Project Consortium, 2015) and restricted to high-quality indels with INFO score (imputation confidence score) > 0.9.

Imputation

We assessed three imputation algorithms intended for low-coverage data: STITCH v1.5.3 (Davies et al., 2016), GLIMPSE v1.0.0 (Davies et al., 2021; Rubinacci et al., 2021), and QUILT v0.1.9 (Davies et al., 2021). For all analyses, OncoPanel data was aligned to hg19 using bwa and processed with the GATK IndelRealigner. The 1000 Genomes Phase 3 release was used as a haplotype reference, targeting variants with > 1% frequency in the European population. Tumor imputation was performed using the 1000 Genomes reference (rather than the HRC reference) because the HRC panel is not publicly available and the HRC imputation server does not support raw sequencing data. We thus sought to use the best reference panels that were accessible for the two data types. We note that HRC largely improves imputation accuracy for low-frequency variants (McCarthy et al., 2016), which were not the target of our analysis.

Imputation with STITCH was carried out on all samples using aligned reads in 5-MB batches (see the “Availability of data and materials” section for the detailed parameters and code). The potential influence of target cohort size was evaluated by randomly downsampling to a lower number of sequenced tumors. Imputation with QUILT was carried out using the same input and batching procedure, with default parameters. Imputation with GLIMPSE was carried out on all samples with default parameters as recommended in the documentation: calling genotype likelihoods from each raw BAM file, splitting the genome into chunks, performing imputation and phasing, and ligating the chunks. An alternative, reference-only version of GLIMPSE was kindly provided to us by the authors but could not be compiled in our computing environment. Lastly, we considered two other imputation approaches: GeneImp (Spiliopoulou et al., 2017) and BEAGLE (Browning et al., 2021), but found that their computational requirements were infeasible for sample sizes in the thousands. Identical reference panel data was used for all methods except small indels, structural variants, and multi-allelic polymorphisms were excluded from the STITCH and GLIMPSE analysis (which only allows biallelic single nucleotides). After imputation, variants were considered “filtered” if they had a minor allele frequency > 1% and an INFO score (imputation confidence score) > 0.4 (similar to parameters used previously (S. Liu et al., 2018)).

Ancestry assignment

Samples were projected into genetic ancestry principal components using the weights previously derived by the SNPWEIGHTS software (Chen et al., 2013) for the continental populations. Weights were constructed from the 1000 Genomes reference groups with ancestry from Northern/Western Europe (CEU), Western Africa (YRI), and China (CHB+CHD). In our data, each component was projected independently as a linear combination of the weights and individual sample dosages (using the plink2 “--score” command). Components were then linearly recalibrated by fitting to self-reported race as an outcome (note this linear recalibration is for interpretation purposes only and does not influence the significance of any downstream associations). To estimate ancestry fractions, we uniformly rescaled the African and Asian components to be between 0 and 1 and additionally uniformly scaled the ancestry of each individual to be between 0 and 1.

<u>BioMe</u>

Genotyping and quality control

BioMe participants have been genotyped using Illumina's Global Screening Array (GSA-24 v1). Samples flagged as being contaminated, possibly duplicated, having low coverage, a call rate < 95%, or showing genotype-exome discordance were removed. Sex discordant samples were etiher reconciled after a plate swap resolution or removed. Sample missingness and depth of coverage were calculated using vcftools: mean missingness was 1.24 x 10^{-3}, mean depth of coverage for all samples was 36.4x. Variant missingness and depth of coverage were calculated using vcftools (Danecek et al., 2011): mean missingness rate of 1.24 x 10^{-3}, mean depth of coverage for all coding sites was 36.4x. Sites with HWE P-values < 1e-6 were retained but flagged.

Imputation

Imputation was performed using the 1000G (1000 Genomes Project Consortium, 2015) and TOPMed (Taliun et al., 2021) reference panel, and the software packages Beagle (Browning et al., 2021) and Impute2 (Howie et al., 2009). A filter of r2 > 0.7 was applied. Approximately 31,700 samples and 7,8M variants passed QC and were used in downstream analyses.

Ancestry assignment

We inferred the genetic ancestry following the guidelines of the Pan UKBB (*Quality Control (QC) | Pan UKBB*, n.d.). We performed a PCA using PLINK (Purcell et al., 2007), excluding relatives above 2nd-degree (kinship method, estimated using KING (Manichaikul et al., 2010)) and variants with MAF < 0.05. We trained a random forest classifier to infer the cohort's genetic ancestry using the 1000G labels as reference, removed outliers (by only including the quantiles 0.25-0.90) and participants with mixed ancestry (random forest probability ≤ 0.5). Inferred ancestry: AMR (n=5,336), AFR (n=5,660), EUR (n=7,447), SAS (n=613), and EAS (n=728).

Supplementary figures

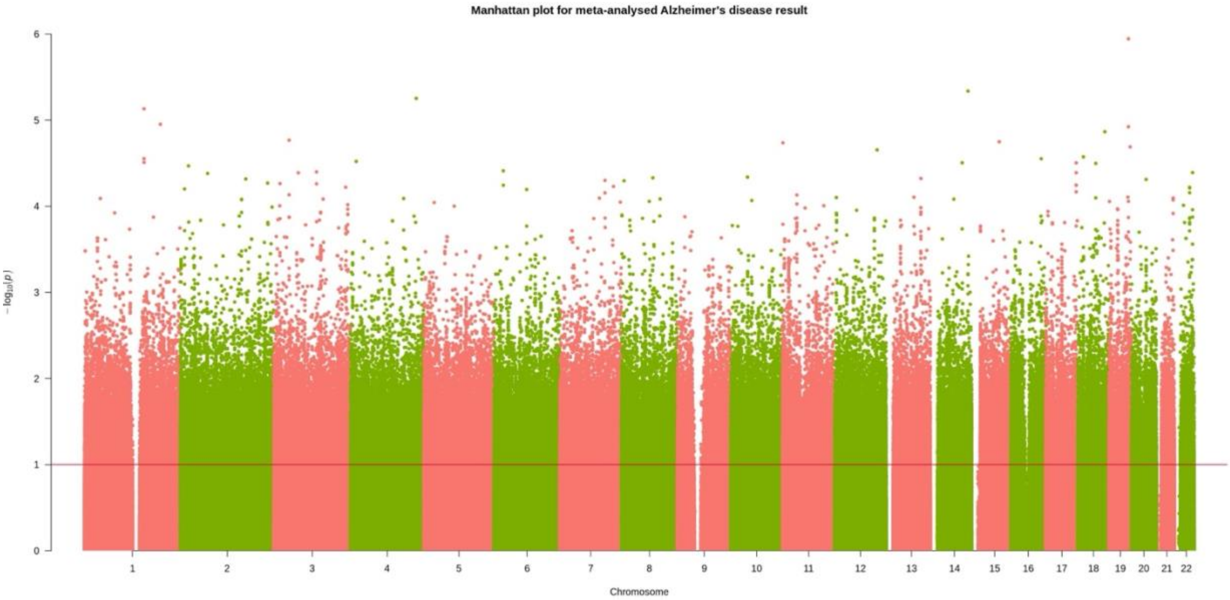

Supplementary Figure 1. Manhattan plot for meta-analyzed Alzheimer's disease mortality GWAS.

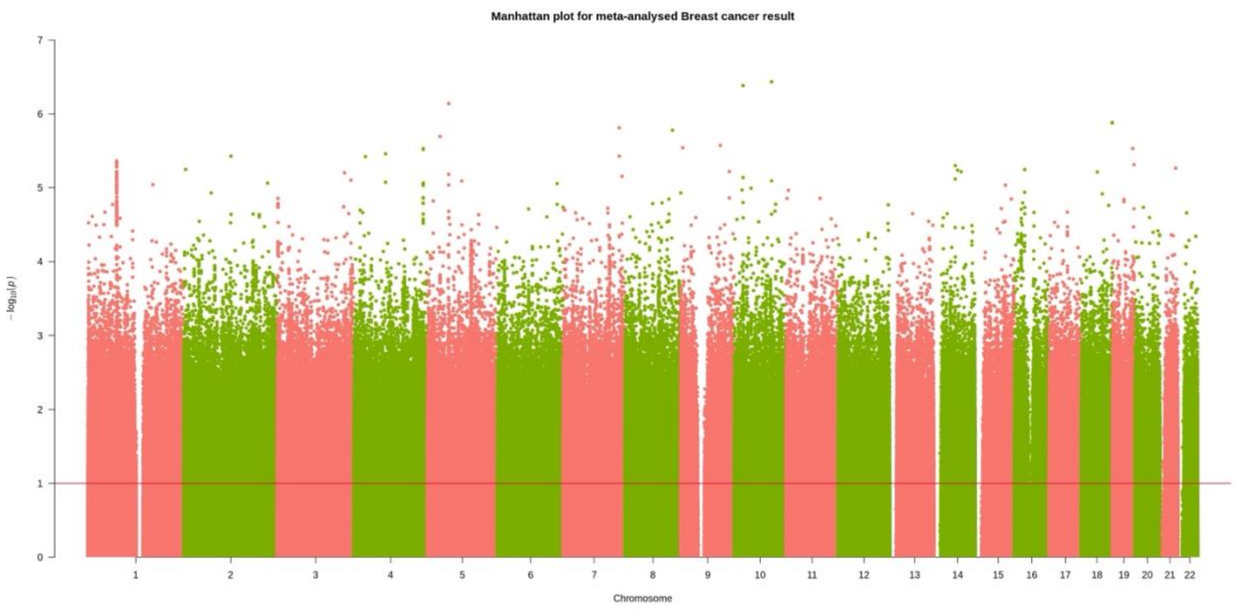

Supplementary Figure 2. Manhattan plot for meta-analyzed breast cancer mortality GWAS.

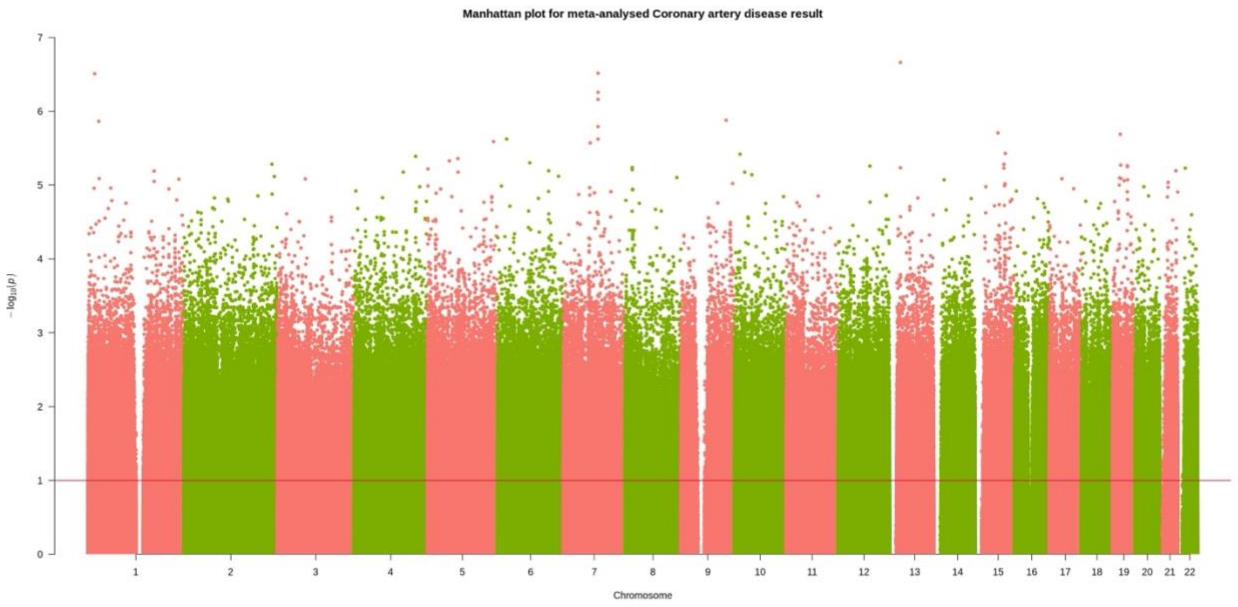

Supplementary Figure 3. Manhattan plot for meta-analyzed coronary artery disease mortality GWAS.

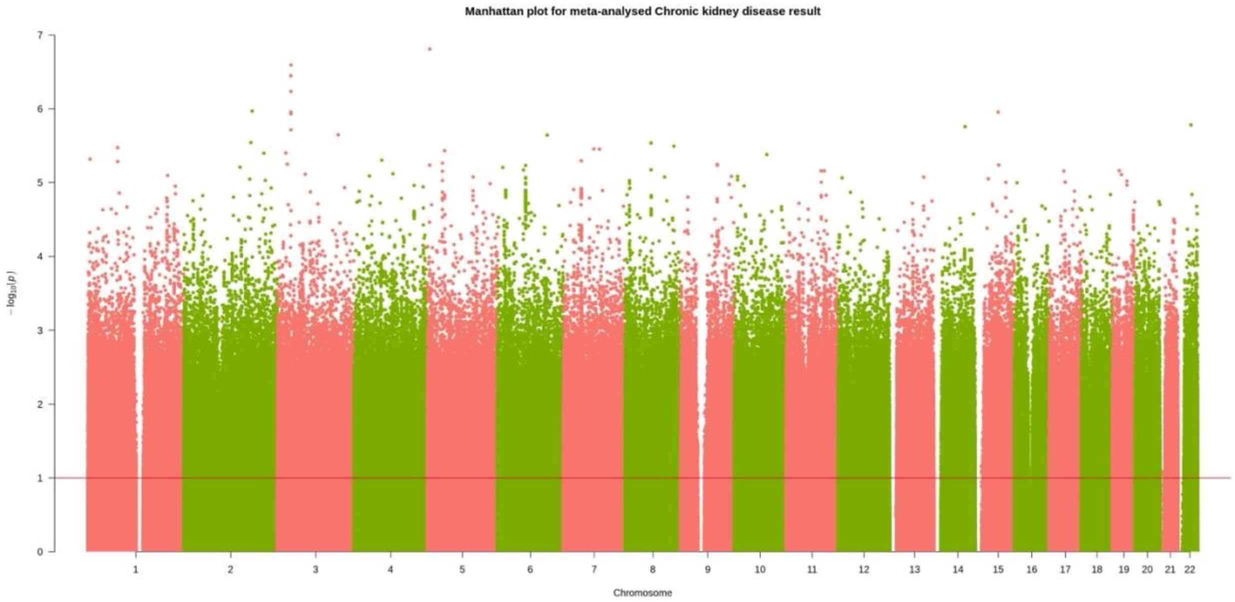

Supplementary Figure 4. Manhattan plot for meta-analyzed chronic kidney disease mortality GWAS.

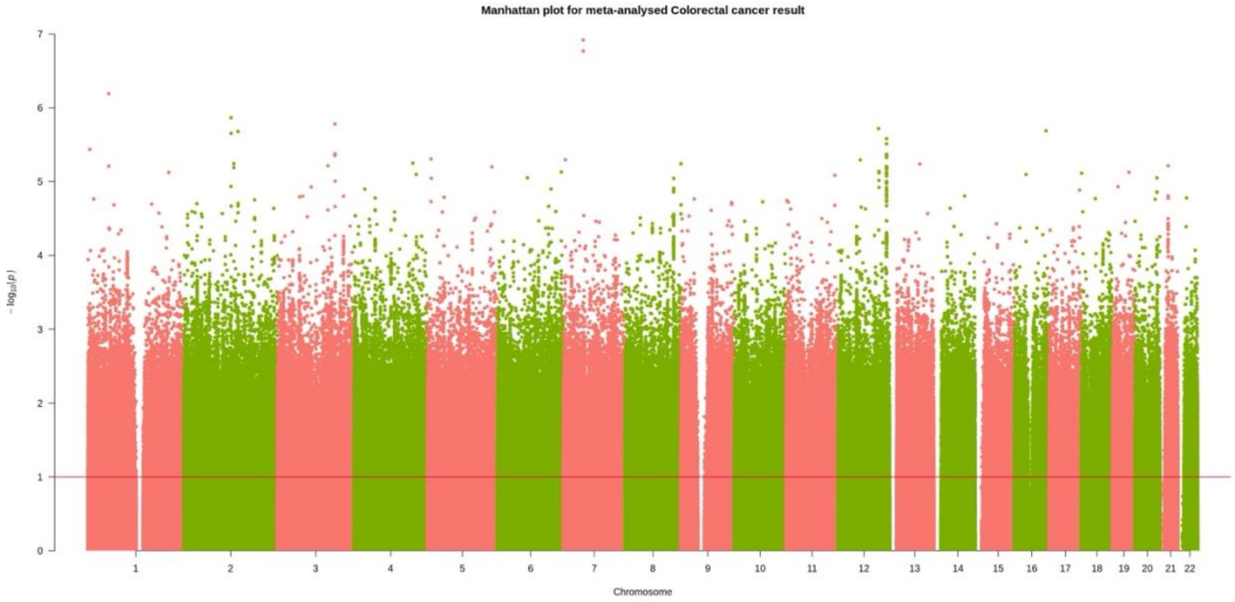

Supplementary Figure 5. Manhattan plot for meta-analyzed colorectal cancer mortality GWAS.

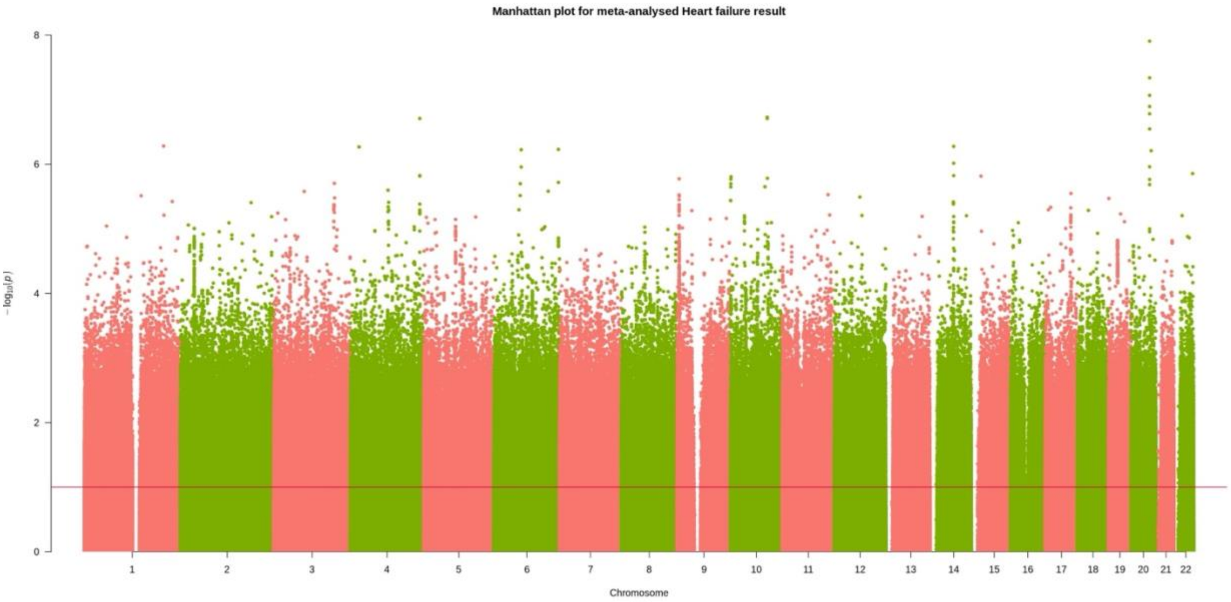

Supplementary Figure 6. Manhattan plot for meta-analyzed heart failure mortality GWAS.

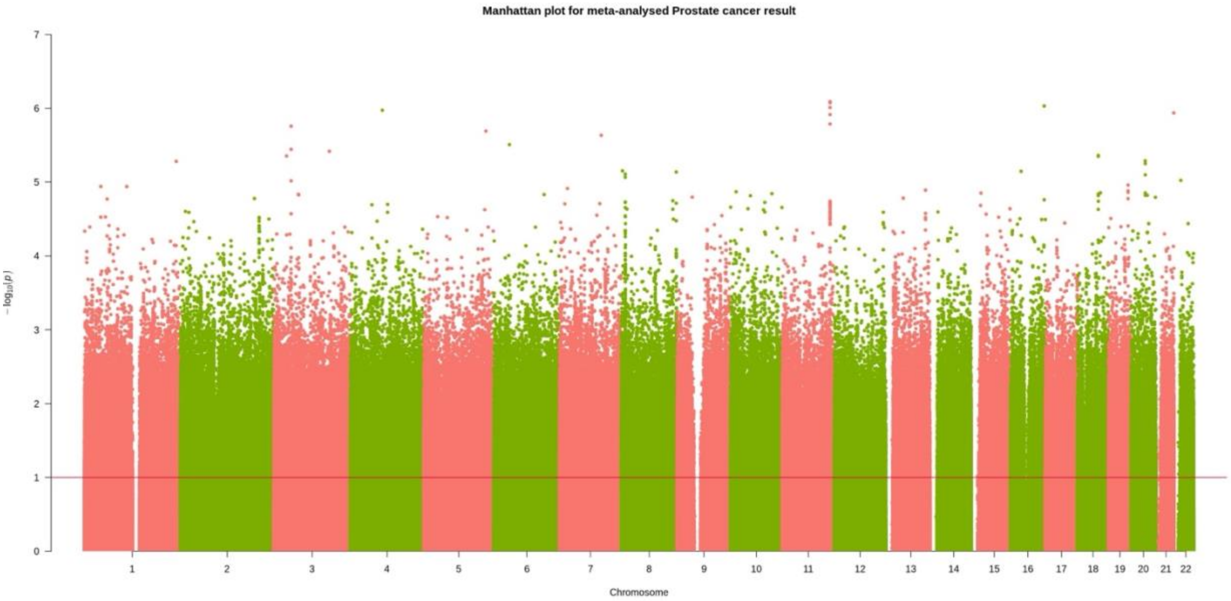

Supplementary Figure 7. Manhattan plot for meta-analyzed prostate cancer mortality GWAS.

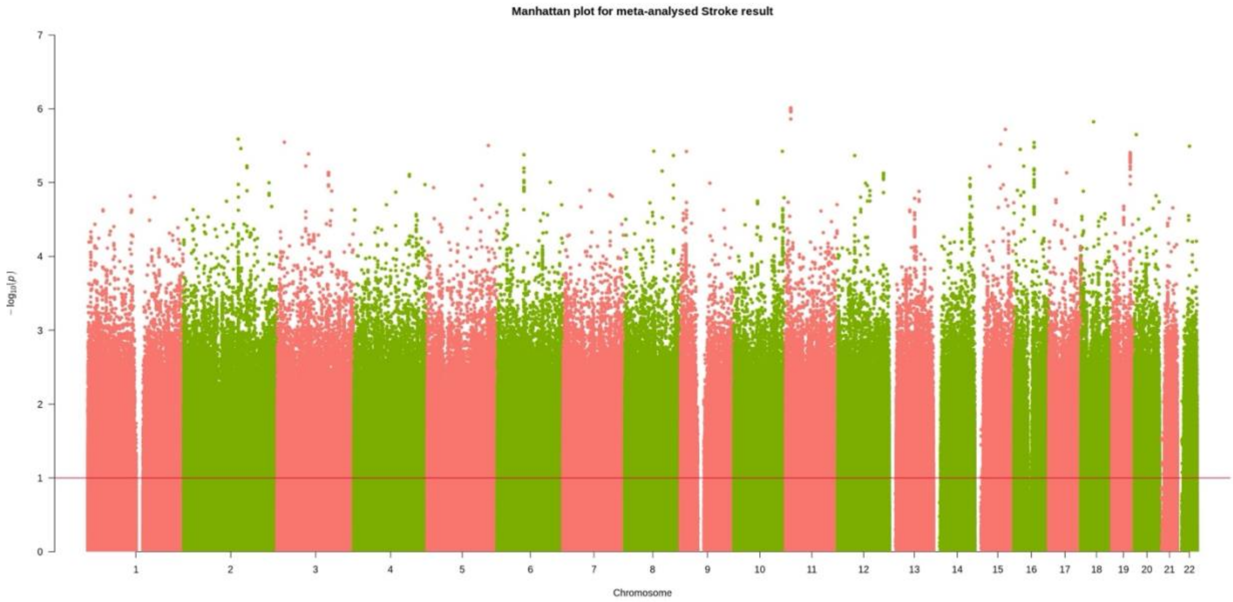

Supplementary Figure 8. Manhattan plot for meta-analyzed stroke mortality GWAS.

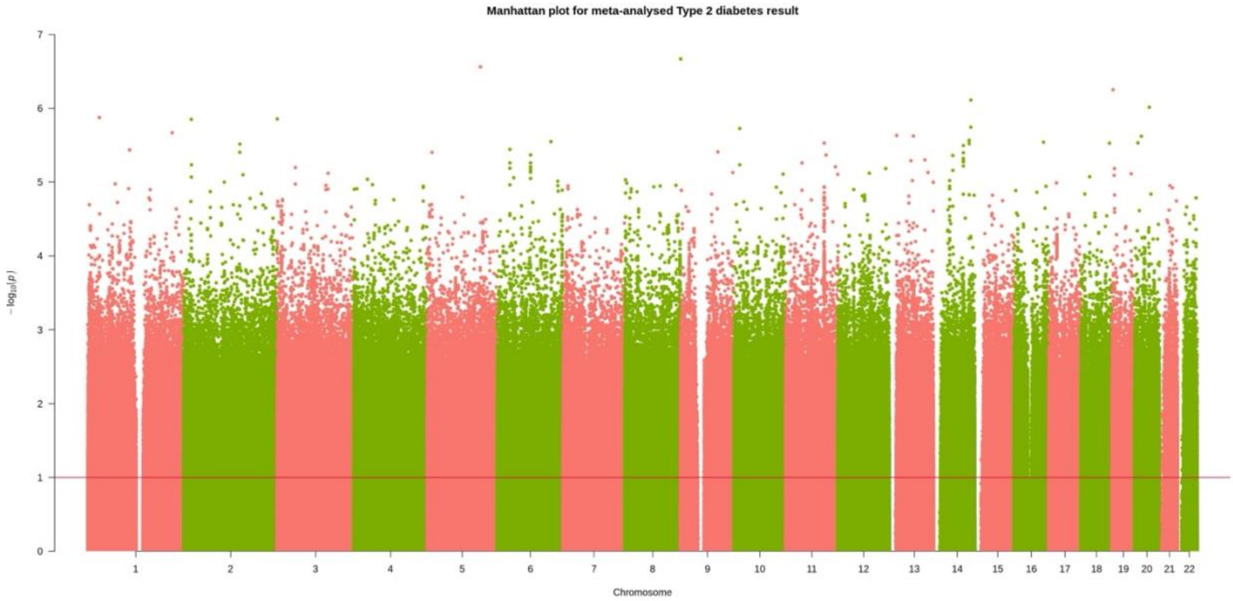

Supplementary Figure 9. Manhattan plot for meta-analyzed type 2 diabetes mortality GWAS.

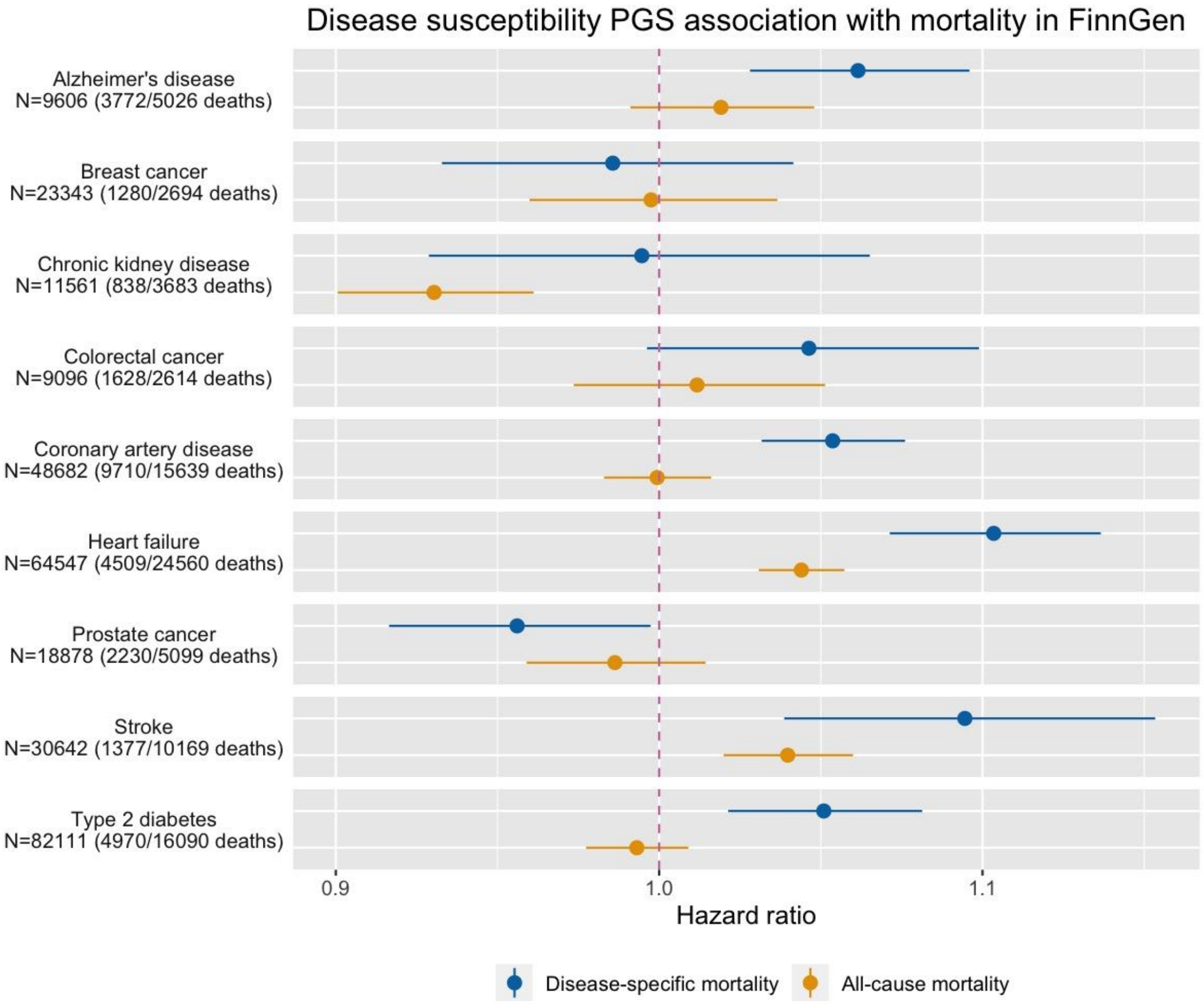

Supplementary Figure 10. Comparison of disease susceptibility PGS association with disease-specific mortality and all-cause mortality in Finngen. In parenthesis stated number of disease-specific mortalities/number of all-cause mortality within the total number of patients (N). Horizontal solid lines represent 95% CI for association HR. Also see **Supplementary Table** 7 for quantitative results.

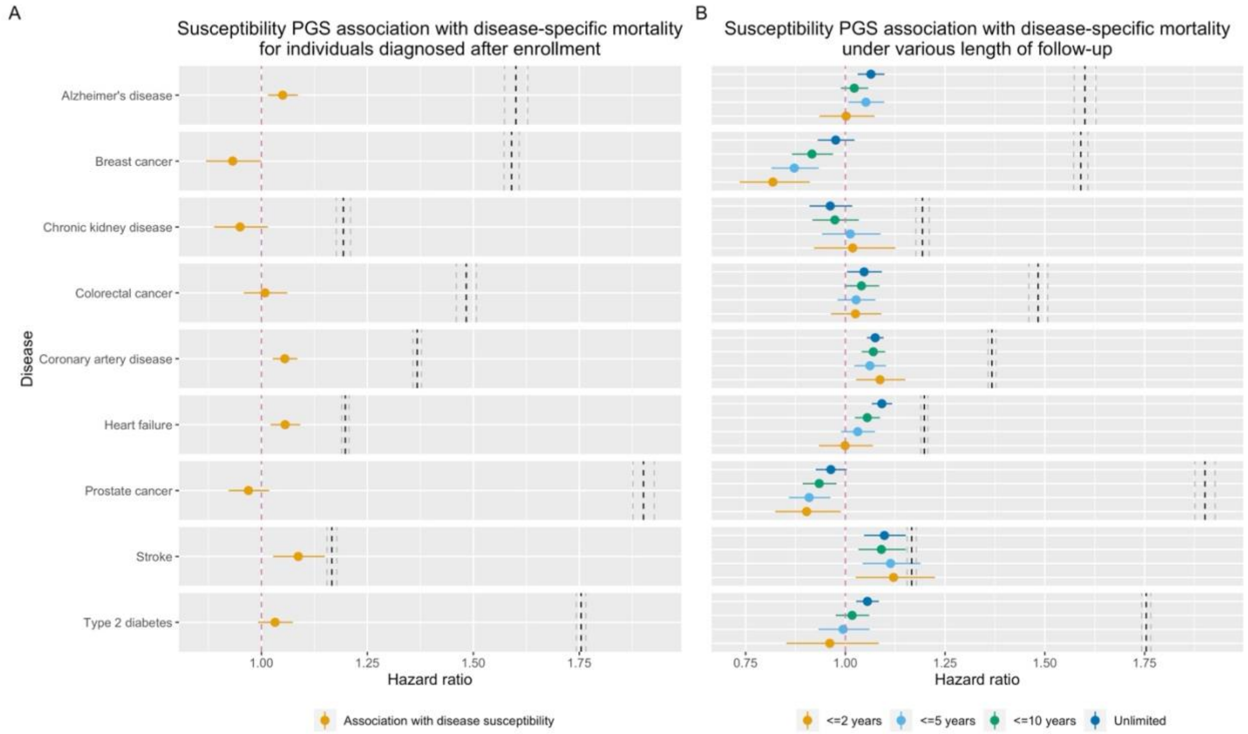

Supplementary Figure 11. Sensitivity analyses. Left: Association between disease susceptibility PGS with disease specific mortality among only patients diagnosed after enrollment; Right: association between disease susceptibility PGS with disease specific mortality among patients with various lengths of follow-up after diagnosis. Horizontal solid lines represent 95% CI for association HR. Also see **Supplementary Table** 8 for quantitative results.

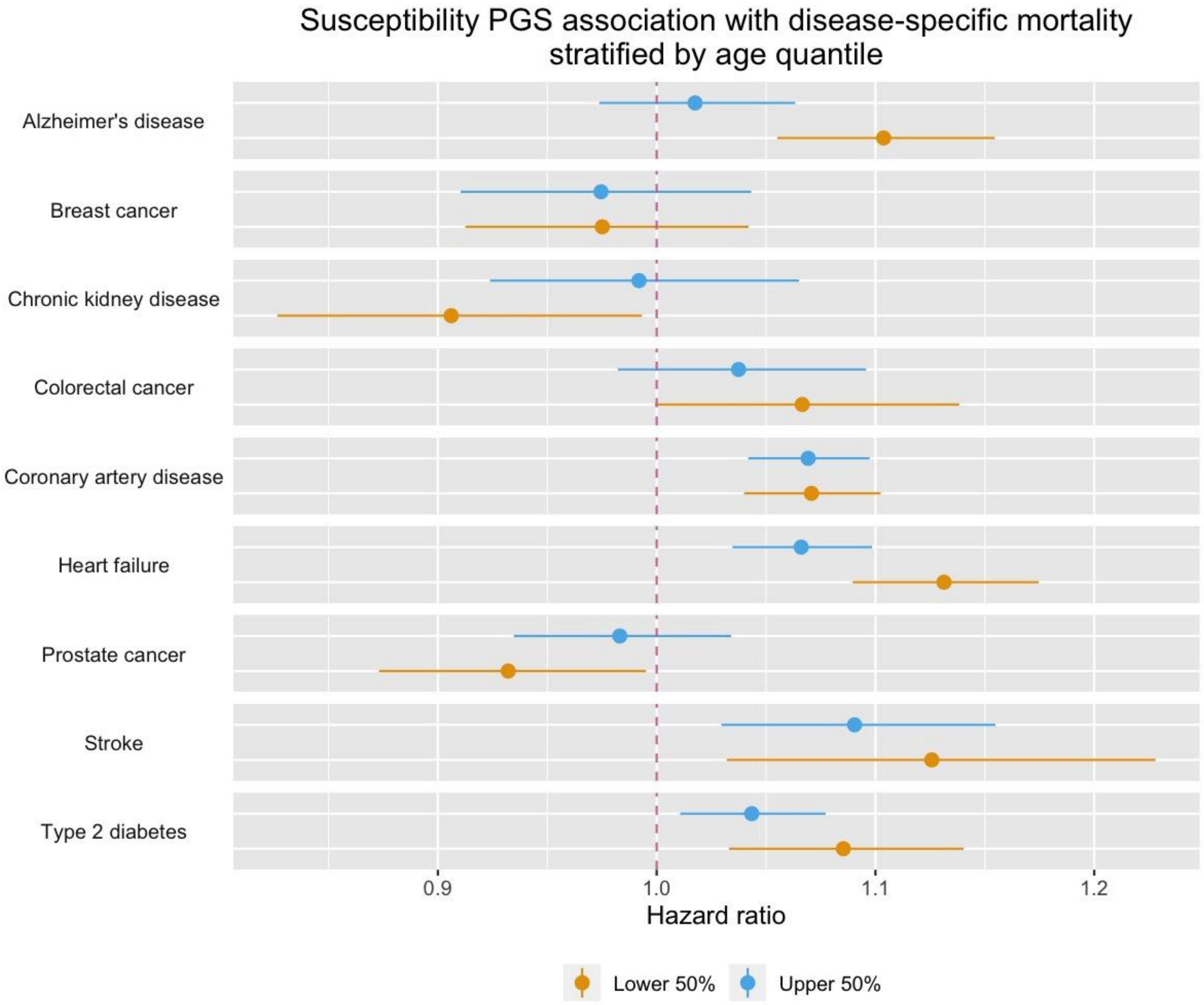

Supplementary Figure 12. Sensitivity analyses. Susceptibility PGS association with disease-specific mortality stratified by age quantile. Horizontal solid lines represent 95% CI for association HR. Also see **Supplementary Table** 9 for quantitative results.

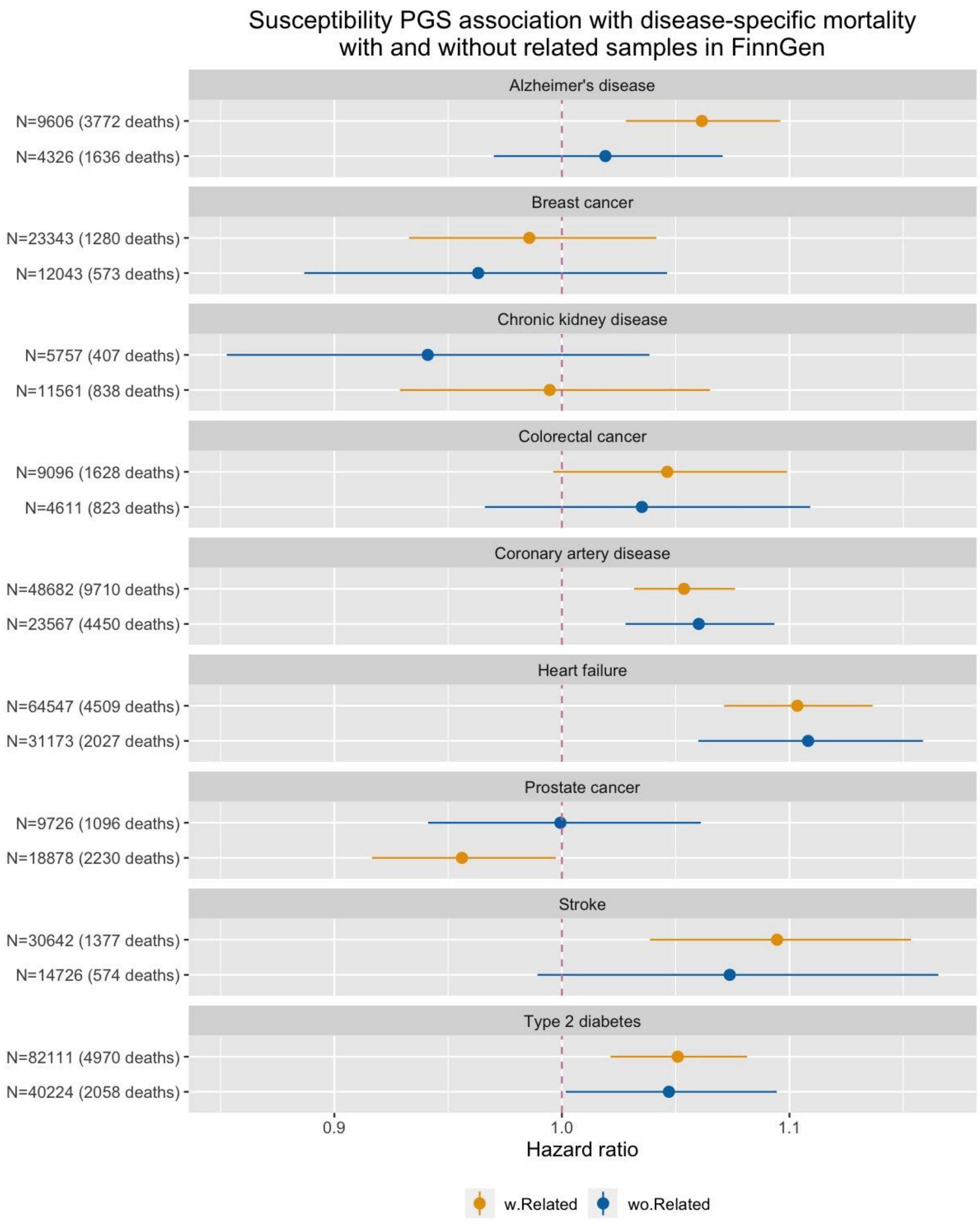

Supplementary Figure 13. Sensitivity analyses. Susceptibility PGS association with disease-specific mortality in FinnGen with and without related individuals. For the without relatedness group (wo.Related), we removed up until second degree relatedness in the analyses. Horizontal solid lines represent 95% CI for association HR. Also see **Supplementary Table** 7 for quantitative results.

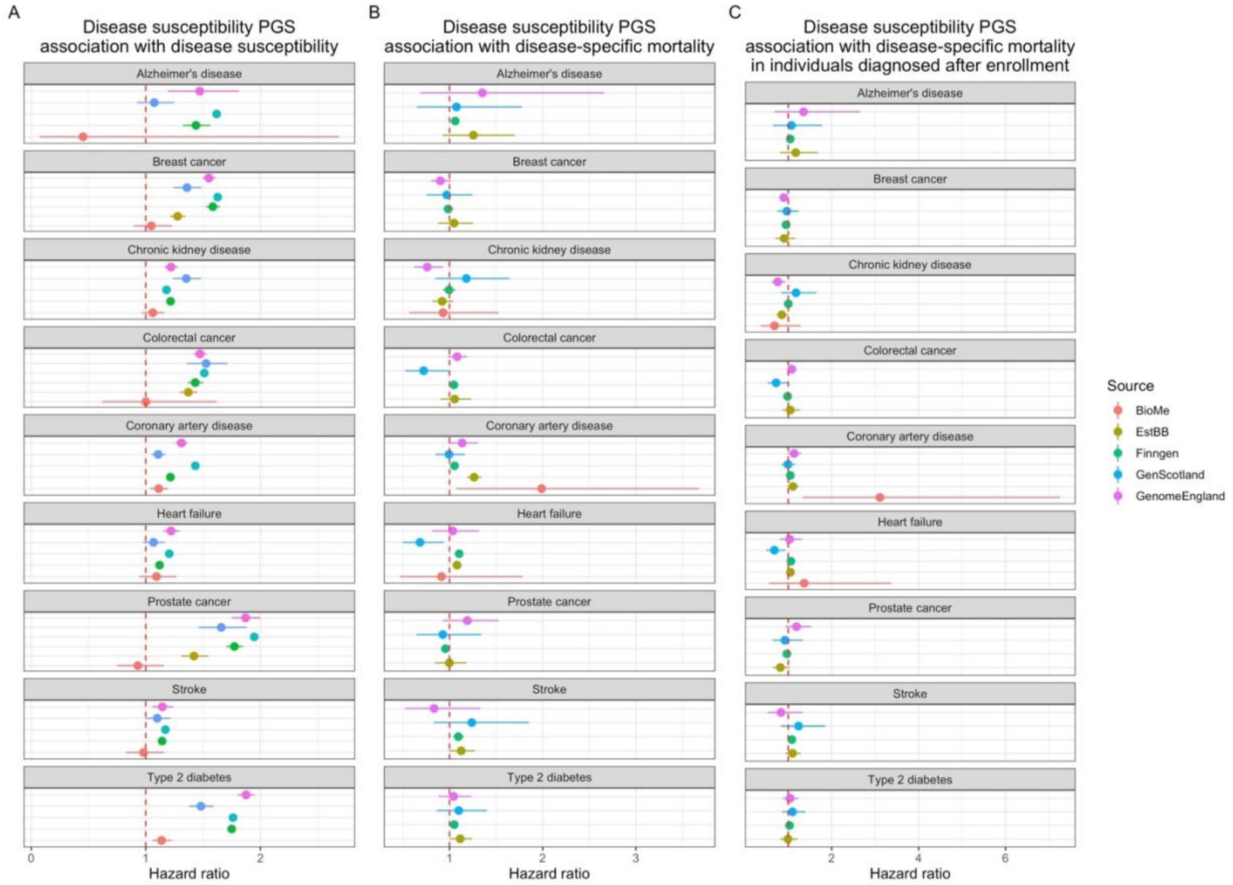

Supplementary Figure 14. Forest plot for effect sizes from each participant biobank. Horizontal solid lines represent 95% CI for association HR.

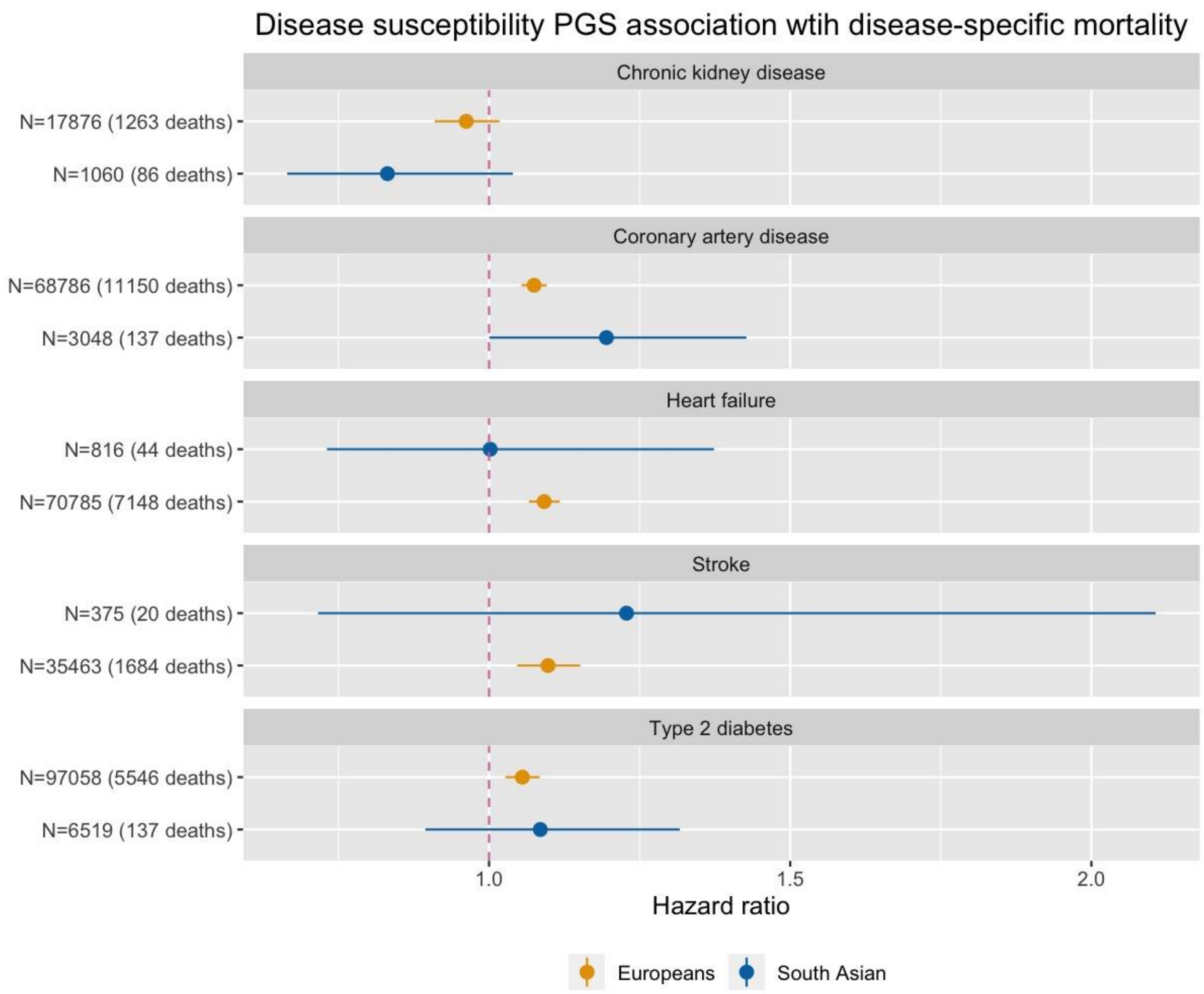

Supplementary Figure 15. Disease susceptibility PGS association with disease-specific mortality in non-European population. As only patient cohorts are of interest in this study, for the non-European population, the only relatively powered results we had were associations for South Asians from biobank Genes & Health in a subset of diseases. Horizontal solid lines represent 95% CI for association HR.

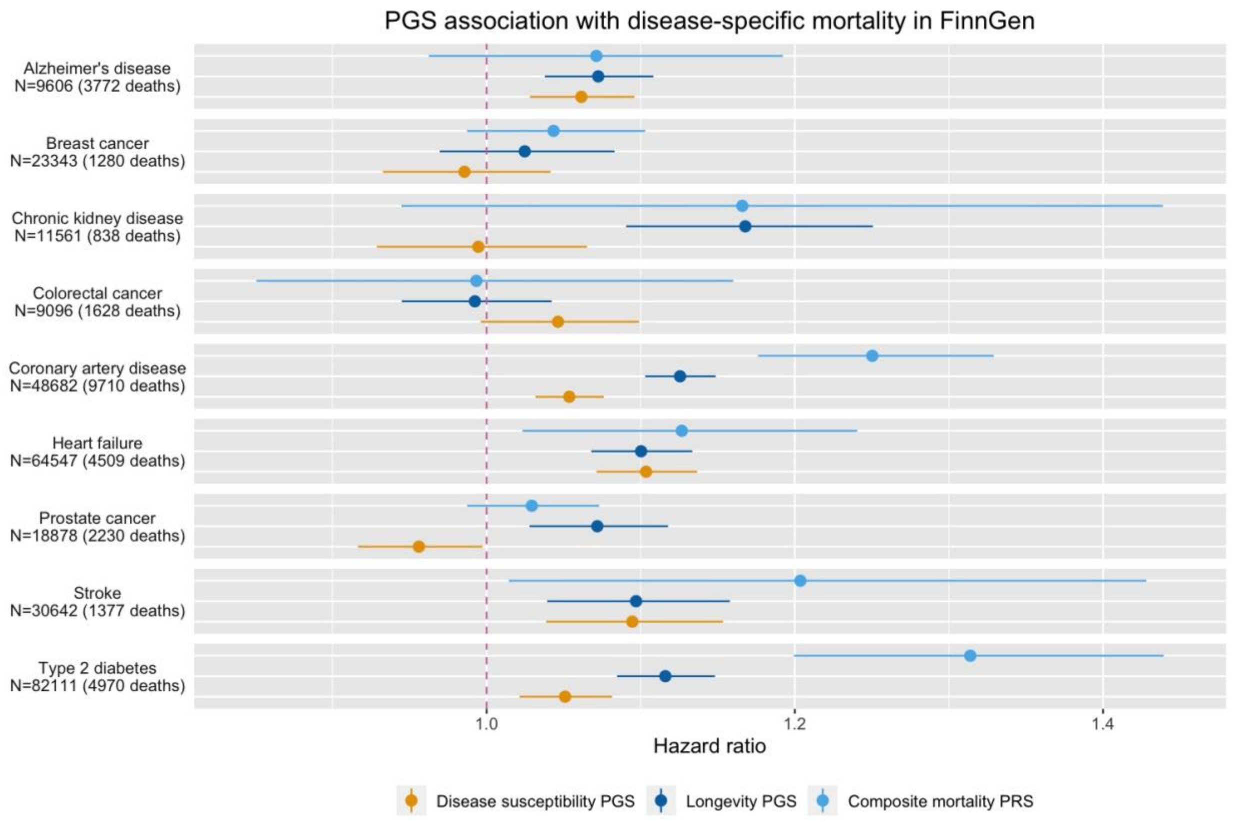

Supplementary Figure 16. Association between a PGS for disease susceptibility (orange dots), longevity (dark blue dots) and composite mortality PGS (light blue dots) with disease-specific mortality in FinnGen. Disease susceptibility PGSs were derived from published large-scale GWAS for each disease. Longevity PGS was derived from (Timmers et al., 2019). Composite mortality PGS was derived from (Meisner et al., 2020). Horizontal solid lines represent 95% CI for association HR. A larger CI is observed for the composite mortality PGS performance due to difference in distribution of male and female PGS (**Supplementary Method**). Also see **Supplementary Table** 12 for quantitative results.

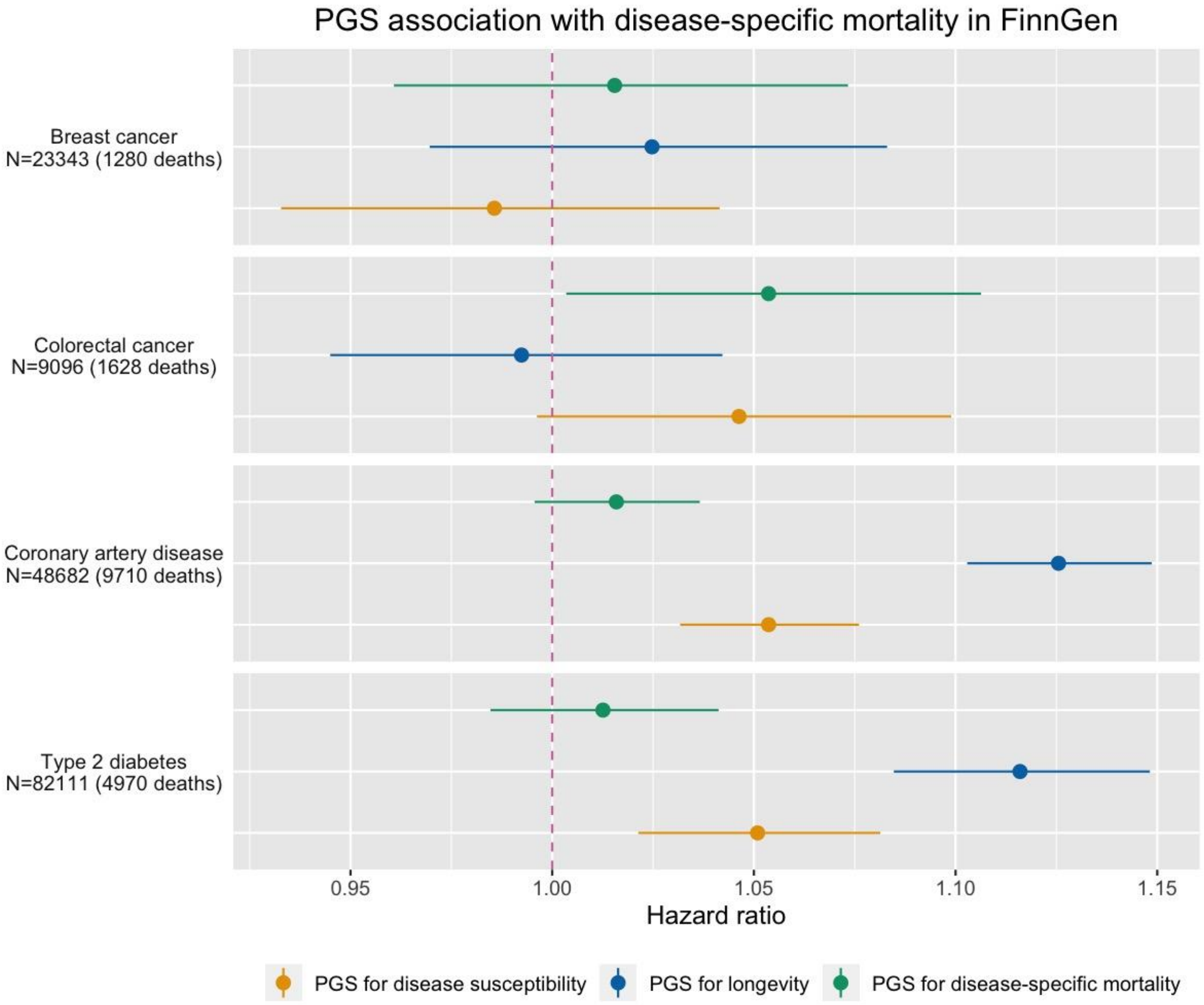

Supplementary Figure 17. Association between PGS and disease-specific mortality in Finngen for eligible diseases. We constructed disease mortality PGS using meta-analyzed mortality GWAS results with Finngen left out and evaluated its association with disease specific mortality in Finngen, comparing with disease diagnosis PGS and longevity PGS. Horizontal solid lines represent 95% CI for association HR. Also see **Supplementary Table** 10 for quantitative results.

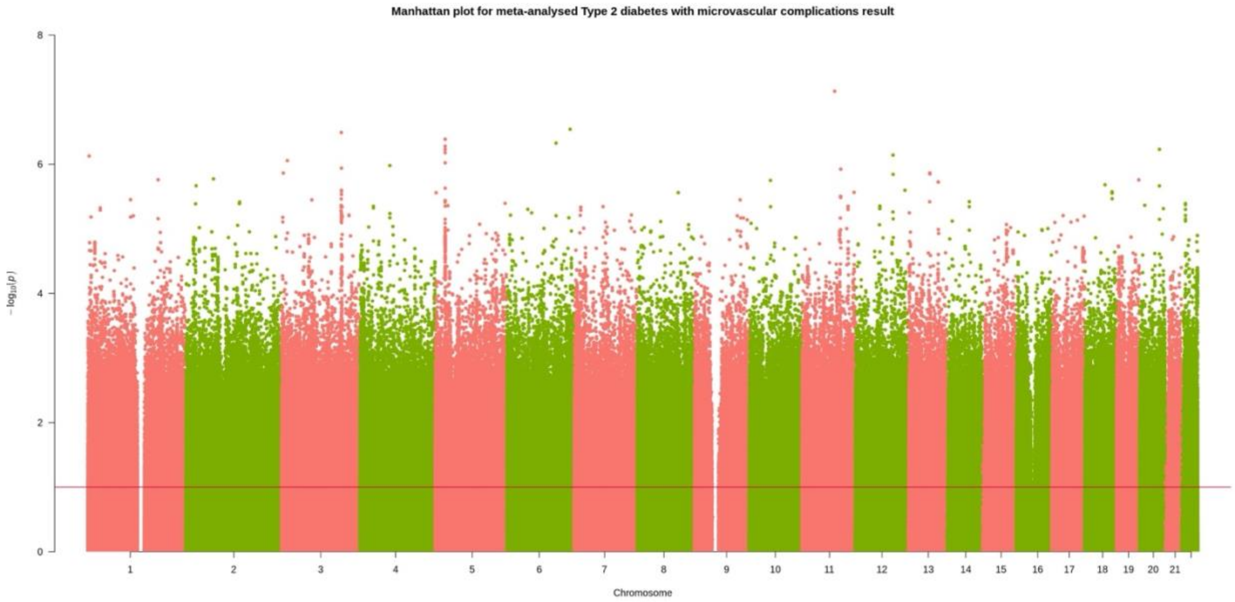

Supplementary Figure 18. Manhattan plot for meta-analyzed type 2 diabetic microvascular complication GWAS.

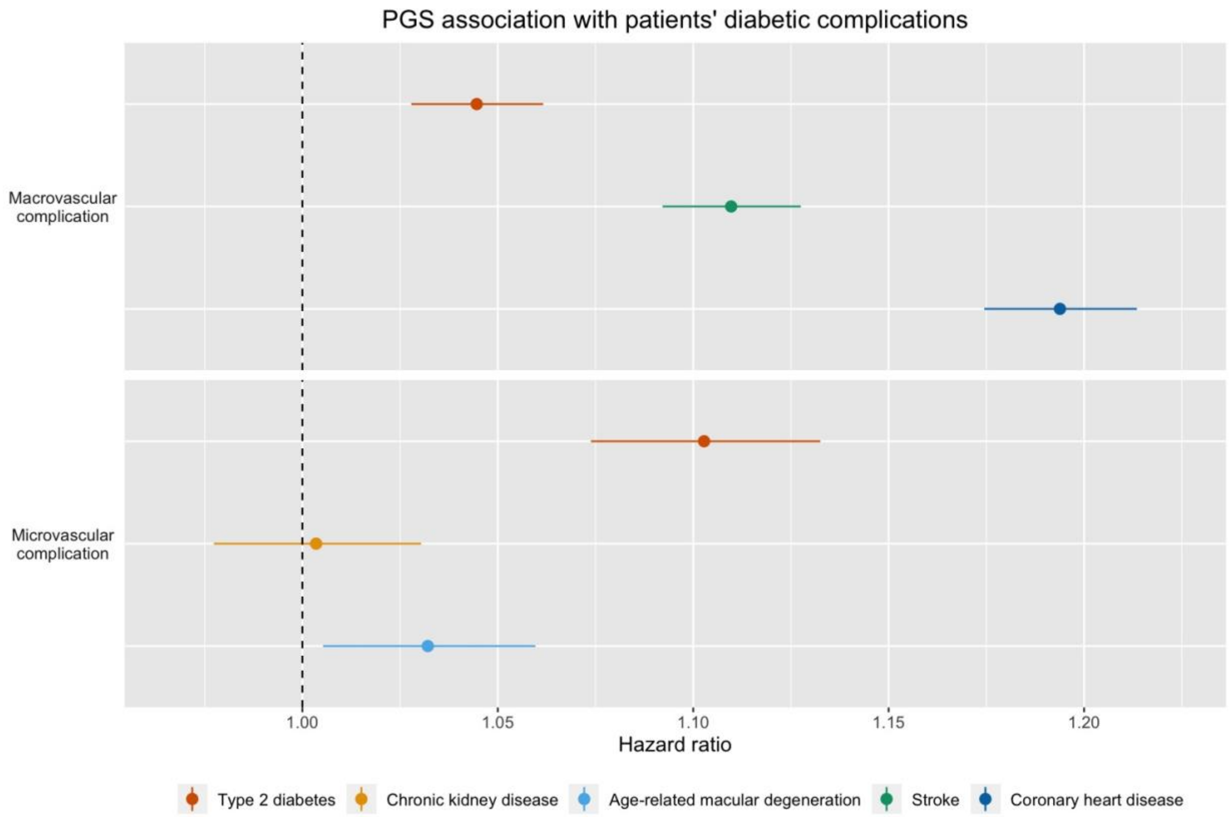

Supplementary Figure 19. Association between various relevant PGS and type 2 diabetic complications. Horizontal solid lines represent 95% CI for association HR. Also see **Supplementary Table** 6 for quantitative results

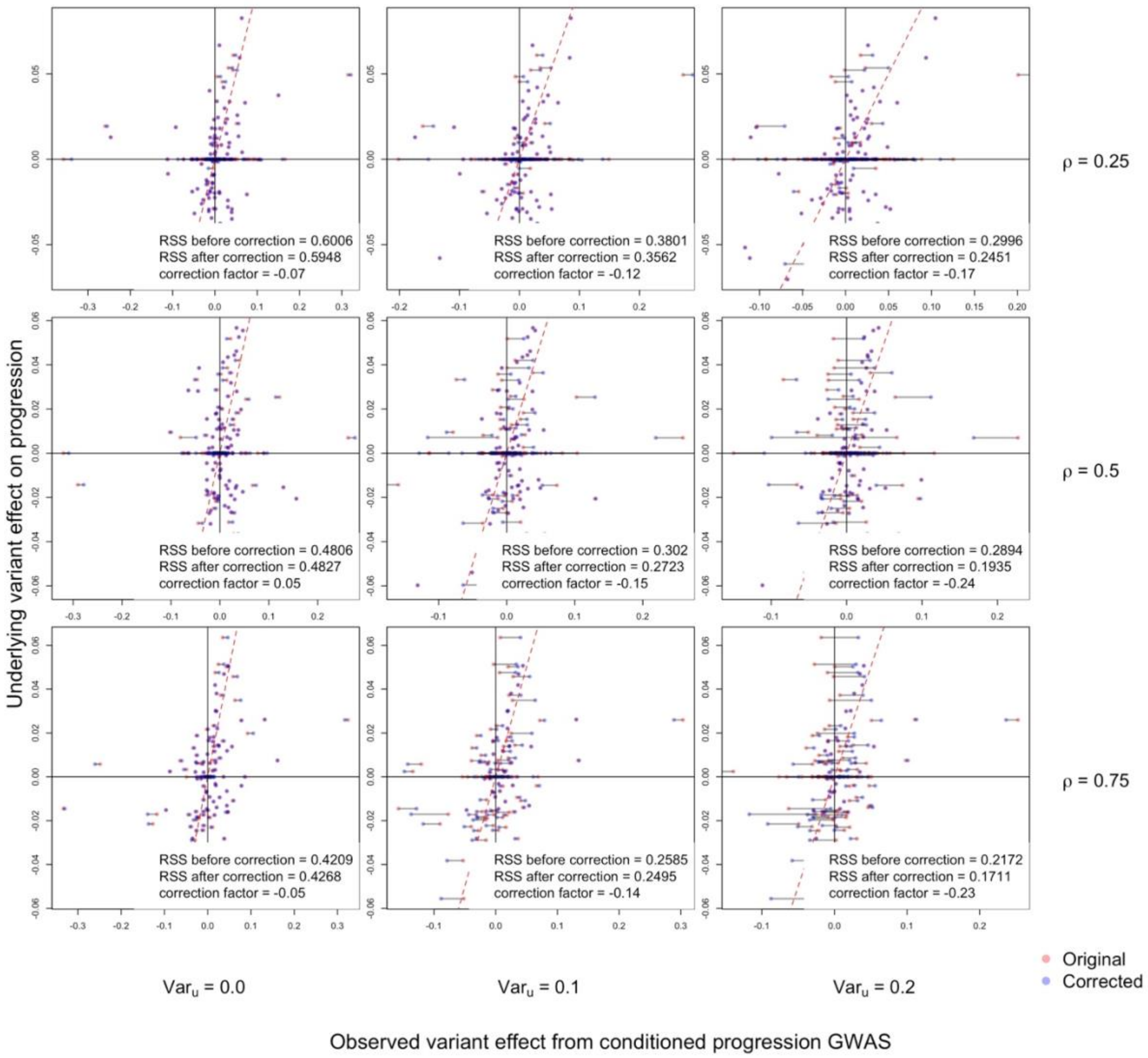

Supplementary Figure 20. Impact of index event bias and slope-hunter-like correction under various conditions. In this experiment, we fixed heritability of disease susceptibility (h_{sus} = 0.2) and progression (h_{out} = 0.005). Impact of susceptibility liability on disease progression liability was also fixed at c = 0.3. Each panel corresponds to a scenario under certain amount of shared causal variants (ρ) and amount of shared non-genetic factor between the two endpoints (Var_u). Plot shows alignment of GWAS observed variant effects (x-axis) with underlying causal effects (y-axis) on disease progression for all causal SNPs before and after slope-hunter-like correction on shared and susceptibility specific causal variants (note susceptibility specific causal variants are on y = 0 axis since their underlying effects on progression are 0). Also see **Supplementary Table** 11 for quantitative results.
