# Supplementary material for: Limited overlap between genetic effects on disease susceptibility and disease survival
Source: Nat Genet. 2025 Sep 30;57(10):2418–26. doi: 10.1038/s41588-025-02342-8 (PMC12513829; doi:10.1038/s41588-025-02342-8)
Supplement: Supplementary file 2 — Reporting Summary [file 41588_2025_2342_MOESM2_ESM.pdf]

nature portfolio

Corresponding author(s): Andrea Ganna

Last updated by author(s): Jun 6, 2025

Reporting Summary

Nature Portfolio wishes to improve the reproducibility of the work that we publish. This form provides structure for consistency and transparency in reporting. For further information on Nature Portfolio policies, see our Editorial Policies and the Editorial Policy Checklist.

Statistics

For all statistical analyses, confirm that the following items are present in the figure legend, table legend, main text, or Methods section.

n/a	Confirmed	
☐	☒	The exact sample size (*n*) for each experimental group/condition, given as a discrete number and unit of measurement
☐	☒	A statement on whether measurements were taken from distinct samples or whether the same sample was measured repeatedly
☐	☒	The statistical test(s) used AND whether they are one- or two-sided<br>*Only common tests should be described solely by name; describe more complex techniques in the Methods section.*
☐	☒	A description of all covariates tested
☐	☒	A description of any assumptions or corrections, such as tests of normality and adjustment for multiple comparisons
☐	☒	A full description of the statistical parameters including central tendency (e.g. means) or other basic estimates (e.g. regression coefficient) AND variation (e.g. standard deviation) or associated estimates of uncertainty (e.g. confidence intervals)
☐	☒	For null hypothesis testing, the test statistic (e.g. *F*, *t*, *r*) with confidence intervals, effect sizes, degrees of freedom and *P* value noted<br>*Give P values as exact values whenever suitable.*
☒	☐	For Bayesian analysis, information on the choice of priors and Markov chain Monte Carlo settings
☐	☒	For hierarchical and complex designs, identification of the appropriate level for tests and full reporting of outcomes
☐	☒	Estimates of effect sizes (e.g. Cohen's *d*, Pearson's *r*), indicating how they were calculated

Our web collection on statistics for biologists contains articles on many of the points above.

Software and code

Policy information about availability of computer code

Data collection	See supplementary notes for biobank specific data collection approach.
Data analysis	Major tools used in data analysis include: PLINK v2, GATE v0.45, SPACox v2.1.0, MegaPRS v5.1, LDSC (LD SCore) v1.0.1, and METAL (version 2011-03-25).

For manuscripts utilizing custom algorithms or software that are central to the research but not yet described in published literature, software must be made available to editors and reviewers. We strongly encourage code deposition in a community repository (e.g. GitHub). See the Nature Portfolio guidelines for submitting code & software for further information.

Data

Policy information about availability of data

All manuscripts must include a data availability statement. This statement should provide the following information, where applicable:

- Accession codes, unique identifiers, or web links for publicly available datasets
- A description of any restrictions on data availability
- For clinical datasets or third party data, please ensure that the statement adheres to our policy

Summary statistics for mortality GWAS of phenotypes of interest can be found in figshare public project https://figshare.com/projects/Progression_GWAS/252002
PGS weights and analyses code can be found in public github repo https://github.com/Zhiyu-9668/ProgressAnalysis

Research involving human participants, their data, or biological material

Policy information about studies with human participants or human data. See also policy information about sex, gender (identity/presentation), and sexual orientation and race, ethnicity and racism.

Reporting on sex and gender	Biological sex has been used as a covariate in GWAS and PGS related analysis. Gender is not of interest in this study.
Reporting on race, ethnicity, or other socially relevant groupings	Race group in the study was determined using PCA or comparable method. Analysis was stratified by grouped race due to cross-group heterogeneity.
Population characteristics	Cohort specific disease and mortality information can be found in supplementary table 2.
Recruitment	Recruitment strategy varies across participating biobanks. We only consider disease onset after enrollment whenever possible to take into account of possible survivor bias.
Ethics oversight	The study was conducted in compliance with the relevant ethical guidelines and approved by the appropriate ethics committees. Ethics approval for the UK Biobank study was obtained from the North West Centre for Research Ethics Committee (11/NW/0382). Ethical approval for the GS:SFHS study was obtained from the Tayside Committee on Medical Research Ethics (on behalf of the National Health Service). Ethical approval for the GS:3D study was obtained from the Tayside Committee on Medical Research Ethics (on behalf of the National Health Service). Ethical approval for the GS:21CGH study was obtained from the Scotland A Research Ethics Committee. Ethical approval for the GS:SFHS study was obtained from the Tayside Committee on Medical Research Ethics (on behalf of the National Health Service). Ethical approval for the GS:3D study was obtained from the Tayside Committee on Medical Research Ethics (on behalf of the National Health Service). Ethical approval for the GS:21CGH study was obtained from the Scotland A Research Ethics Committee.Ethical approval for the GS:SFHS study was obtained from the Tayside Committee on Medical Research Ethics (on behalf of the National Health Service). Ethical approval for the GS:3D study was obtained from the Tayside Committee on Medical Research Ethics (on behalf of the National Health Service). Ethical approval for the GS:21CGH study was obtained from the Scotland A Research Ethics Committee.

Note that full information on the approval of the study protocol must also be provided in the manuscript.

Field-specific reporting

Please select the one below that is the best fit for your research. If you are not sure, read the appropriate sections before making your selection.

☒ Life sciences ☐ Behavioural & social sciences ☐ Ecological, evolutionary & environmental sciences

For a reference copy of the document with all sections, see nature.com/documents/nr-reporting-summary-flat.pdf

Life sciences study design

All studies must disclose on these points even when the disclosure is negative.

Sample size	We use the maximum available samples after quality control across all partnership.
Data exclusions	We consider only patients having a follow-up duration >= 3mo. after being diagnosed with each disease of interest to grant enough data coverage for included individuals. Depending on biobank recruitment strategy, we also consider only individuals diagnosed after enrollment whenever possible to take into account of possible survivor bias.
Replication	Not applicable since no robust signal from mortality GWAS to be replicated.
Randomization	Phenotypes from each biobank are defined using ICD code as listed in supplementary table 2. Randomization does not apply to this study design.
Blinding	Our study was not a controlled trial but a genome-wide association study (GWAS). Blinding and randomization do not apply.

Reporting for specific materials, systems and methods

We require information from authors about some types of materials, experimental systems and methods used in many studies. Here, indicate whether each material, system or method listed is relevant to your study. If you are not sure if a list item applies to your research, read the appropriate section before selecting a response.

Materials & experimental systems

n/a	Involved in the study
☒	☐ Antibodies
☒	☐ Eukaryotic cell lines
☒	☐ Palaeontology and archaeology
☒	☐ Animals and other organisms
☒	☐ Clinical data
☒	☐ Dual use research of concern
☒	☐ Plants

Methods

n/a	Involved in the study
☒	☐ ChIP-seq
☒	☐ Flow cytometry
☒	☐ MRI-based neuroimaging

Plants

Seed stocks	NA
Novel plant genotypes	NA
Authentication	NA
